# Genetic mutations linked to field‐evolved Cry1Fa-resistance in the European corn borer, *Ostrinia nubilalis*

**DOI:** 10.1038/s41598-023-35252-y

**Published:** 2023-05-18

**Authors:** Yasmine Farhan, Jocelyn L. Smith, Michael G. Sovic, Andrew P. Michel

**Affiliations:** 1 Department of Plant Agriculture, University of Guelph, Ridgetown Campus, Ridgetown, ON Canada; 2 Infectious Diseases Institute, The Ohio State University, Pickerington, OH USA; 3 Department of Entomology, The Ohio State University, Wooster, OH USA

**Keywords:** Evolution, Molecular biology, Genetics, Agricultural genetics, Genetic markers

## Abstract

Transgenic corn, *Zea mays* (L.), expressing insecticidal toxins such as Cry1Fa, from *Bacillus thuringiensis* (Bt corn) targeting *Ostrinia nubilalis* (Hübner) (Lepidoptera: Crambidae) resulted in over 20 years of management success. The first case of practical field-evolved resistance by *O. nubilalis* to a Bt corn toxin, Cry1Fa, was discovered in Nova Scotia, Canada, in 2018. Laboratory-derived Cry1Fa-resistance by *O. nubilalis* was linked to a genome region encoding the ATP Binding Cassette subfamily C2 (*ABCC2*) gene; however, the involvement of *ABCC2* and specific mutations in the gene leading to resistance remain unknown. Using a classical candidate gene approach, we report on *O. nubilalis ABCC2* gene mutations linked to laboratory-derived and field-evolved Cry1Fa-resistance. Using these mutations, a DNA-based genotyping assay was developed to test for the presence of the Cry1Fa-resistance alleles in *O. nubilalis* strains collected in Canada. Screening data provide strong evidence that field-evolved Cry1Fa-resistance in *O. nubilalis* maps to the *ABCC2* gene and demonstrates the utility of this assay for detecting the Cry1Fa resistance allele in *O. nubilalis*. This study is the first to describe mutations linked to Bt resistance in *O. nubilalis* and provides a DNA-based detection method that can be used for monitoring.

## Introduction

Genes encoding insecticidal toxins from the bacterium *Bacillus thuringiensis* (Bt) Berliner were engineered into crops to safely and sustainably manage insect pests^1^. Since their first year of commercialization in 1996, the global area planted to Bt crops increased from 1.1 million to 109 million hectares in 2019^1^. Intoxication with Bt toxins follows a series of steps that involve activation of the toxin by midgut proteases, binding of the activated toxin to receptors on the brush membrane of the midgut, and formation of pores that result in feeding cessation followed by death^2–4^. Changes to these steps can result in Bt resistance, which is a genetically based decrease in susceptibility of an insect population to a Bt toxin^4,5^.

Cases of field-evolved resistance to Bt crops increased from three in 2005 to 43 in 2020^5^. The cases of field-evolved Bt resistance have involved 15 pest species and ten Bt toxins in nine countries^5^; yet, the mechanisms of resistance and causal genetic mutations are known only for *Spodoptera frugiperda* (J. E. Smith) with field-evolved Cry1F resistance and *Pectinophora gossypiella* (Saunders) with field-evolved Cry1Ac and Cry2Ab resistance^6–10^. The first case of practical field-evolved resistance to Bt corn, *Zea mays* L., by *O. nubilalis* was discovered in 2018 in Nova Scotia, Canada, involving Cry1Fa^11^. Two additional Cry1Fa-resistant populations have since been detected in Quebec and Manitoba^12^. Early warning signs of resistance to Cry1Ab, Cry1A.105, and Cry2Ab2 toxins have also been detected in eastern Canada^12^. These findings suggest that Cry1Fa resistance may be spreading, and additional *O. nubilalis* resistance issues may be on the rise. Understanding the mechanisms involved in field-evolved Bt resistance may help in the development of much needed DNA-based monitoring and detection methods.

Understanding of the genetics of Bt resistance has primarily come from studies on laboratory-derived Bt resistance which may differ from field-evolved resistance. The most commonly described method of Bt resistance is a change in toxin binding to midgut receptors^4^. Mutations in ATP binding cassette (*ABC*) genes have been linked to resistance against Cry1, Cry2, and Cry3 toxins^6,7,13–21^. In the case of Cry1Fa, resistance has been linked to the *ABC* subfamily *C* member *2* (*ABCC2*) gene in many lepidopteran species such as *Bombyx mori* (L.), *Spodoptera frugiperda* (J. E. Smith), *O. furnacalis* (Guenée), and *O. nubilalis*^6,7,13–18^. Furthermore, Vellichirammal et al. found genes likely involved in Bt toxin mode of action such as *ABCC2*, aminopeptidase, and several other serine proteases were down regulated in Cry1Fa susceptible but not in laboratory-derived Cry1Fa-resistant *O. nubilalis* when exposed to Cry1Fa toxin^18^; however, these genes were not investigated further. The *ABCC2* gene produces a transmembrane protein that typically consists of two transmembrane domains, connected by extracellular and intracellular loops (ECL and ICL, respectively), and two nucleotide binding domains (NBD). Mutations to *ABCC2* causing Cry toxin resistance include nucleotide insertions, substitutions, and deletions, resulting in changes to ECL 2 and 4, NBD2, and protein truncation^6,7,13–15,22–24^.

Genetic linkage mapping indicated that laboratory-derived Cry1Fa-resistance in *O. nubilalis* was controlled by a single quantitative trait locus and involved a single gene^17,25^. In this study, we combined previous research on *ABCC2* genes with a classic candidate gene approach to investigate field-evolved Cry1Fa-resistance in *O. nubilalis*. The specific aims of this study were to (1) determine the heritability and level of recessivity of the field-evolved Cry1Fa-resistance trait in *O. nubilalis*, (2) identify mutations in the candidate gene linked to field-evolved Cry1Fa-resistance, and (3) develop a DNA-based Cry1Fa resistance monitoring method.

Our results indicate that the field-evolved Cry1Fa-resistance trait in *O. nubilalis* is highly recessive. Sequencing analysis revealed multiple SNPS in the *ABCC2* gene which strongly support *ABCC2* as a causal agent of Cry1Fa resistance. Furthermore, Cry1Fa-resistance in *O. nubilalis* is genetically linked to a single amino acid change in one of the extracellular loops and a protein truncating mutation in a known Cry toxin receptor. This study is the first to describe mutations linked to Bt resistance in *O. nubilalis* and provides a DNA-based detection method that can be used for monitoring. Additionally, this work demonstrates the value and efficacy of candidate gene approaches for identifying mechanisms of resistance, such as Bt resistance in economically important agricultural systems.

## Results

### Toxin-overlay diet bioassays

The susceptibility of four *O. nubilalis* strains to Cry1Fa was determined using a toxin-overlay diet bioassay with a non-treated control (0 ng Cry1Fa cm^−2^) and a diagnostic concentration of 200 ng Cry1Fa cm^−2^. Mortality of ON-S1 and ON-S2 was 100% with the diagnostic concentration, which was significantly greater than the mortality of NS-R and QC-R (< 25%) and the mortality of all strains in the non-treated control (< 15%) (Table 1). There was no difference in mortality between concentrations for NS-R and QC-R (Table 1). Based on these results, ON-S1 and ON-S2 were deemed susceptible to Cry1Fa, while NS-R and QC-R were deemed resistant.

**Table 1:** Mortality of field-derived and back-cross strains of *Ostrinia nubilalis* with and without exposure to a diagnostic concentration of Cry1Fa (200 ng cm^−2^).

Strains	n^1^	Mean mortality (% ± SE)	
		Non-treated control	200 ng Cry1Fa cm^−2^
ON-S1	240	12 (3.3)b^2^	100 (0.0)a
ON-S2	240	11 (3.3)b	100 (0.0)a
NS-R	240	13 (3.5)b	23 (4.7)b
QC-R	240	8 (2.7)b	17 (4.0)b
F_3,32_ = 22.30, P < 0.0001			

^1^Total number of larvae infested in bioassay.

^2^Means with the same letter are not significantly different (Tukey’s HSD test, *p* > 0.05).

### Introgression experiment

An introgression experiment was conducted to establish a back-cross strain that is an isoline of ON-S1 but resistant to Cry1Fa. To do this, ON-S1 was crossed with NS-R1 and the heterozygote F_1_ where then interbred. Following this, F_2_, selected for their resistance to Cry1Fa, were back-crossed with ON-S1. This process was repeated 5 times, as shown in Table 2 and described in materials and methods section. The diagnostic concentration of Cry1Fa caused 100% mortality among the offspring of ON-S1 crossings (ON-S1 × F_n_), but mortality among the interbred crossing (F_n_ × F_n_) was significantly lower, ranging between 70–76% (Table 2). Mortality in all back-crossed strains was significantly greater when exposed to the Cry1Fa diagnostic concentration relative to the non-treated control (Table 2). These results demonstrate that the Cry1Fa resistance allele is recessive and inheritance followed Mendelian expectations for a single gene (Table 2). BC-R was established by interbreeding F_10_ individuals that survived exposure to the Cry1Fa diagnostic concentration. Exposing this strain to the diagnostic concentration of Cry1Fa caused 17% mortality which did not differ from the non-treated control (Table 2).

**Table 2:** Mortality of *Ostrinia nubilalis* larvae from the back-cross experiment with and without exposure to a diagnostic concentration of Cry1Fa (200 ng cm^−2^).

Parental cross	Offspring	Mortality (% ± SE)			
		n^1^	Non-treated control	n^1^	200 ng Cry1Fa cm^−2^
ON-S1 × NS-R	F_1_	120	5 (1.4)c^2^	120	100 (0.0)a
F_1_ × F_1_	F_2_	120	7 (2.8)c	360	74 (3.8)b
ON-S1 × F_2_	F_3_	120	12 (3.4)c	120	100 (0.0)a
F_3_ × F_3_	F_4_	120	6 (2.1)c	300	70 (6.2)b
ON-S1 × F_4_	F_5_	120	8 (2.4)c	120	100 (0.0)a
F_5_ × F_5_	F_6_	120	13 (3.5)c	368	75 (3.8)b
ON-S1 × F_6_	F_7_	120	2 (1.6)c	120	100 (0.0)a
F_7_ × F_7_	F_8_	120	4 (1.9)c	452	75 (4.3)b
ON-S1 × F_8_	F_9_	120	10 (3.2)c	120	100 (0.0)a
F_9_ × F_9_	F_10_	120	7 (2.6)c	360	76 (4.8)b
F_10_ × F_10_^3^	BC-R	120	11 (3.3)c	120	17 (4.1)c
F_10,88_ = 13.49, P < 0.0001					

^1^Total number of larvae infested in bioassay.

^2^Means with the same letter are not significantly different (Tukey’s HSD test, *p* > 0.05).

^3^Interbreeding F_10_ individuals that survived exposure to Cry1Fa diagnostic concentration (200 ng cm^−2^).

### Sequencing

**Figure 1:**
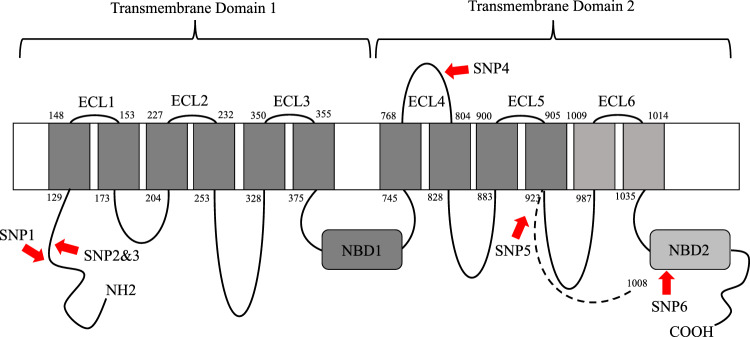
Representative diagram of the predicted Cry1Fa-susceptible ABCC2 protein structure and location of the SNPs in the Cry1Fa-resistant ABCC2 protein in *Ostrinia nubilalis*. Diagram is not drawn to scale, including the length of the extra-cellular loops (ECL) and the localization of the nucleotide binding domain (NBD). The diagram includes the approximate location of the SNPs in Cry1Fa-resistant ABCC2 protein (red arrows). Numbers indicate the amino acid residue at that location. The dotted lines and light grey colour for the Transmembrane Domain 2 represent the predicted region missing in the truncated Cry1Fa-resistant ABCC2 protein.

The *ABCC2* gene of *O. nubilalis* contained 24 predicted exons, totaling 4,089 bp of coding sequence. We sequenced ~ 4.0 Kb (> 98%) of the *ABCC2* coding region from ON-S1 and NS-R. In total, we found 20 SNPs (~ 1 per 200 bp) between resistant and susceptible individuals and relative to the reference gene. Most SNPs were synonymous substitutions that did not alter the amino acid sequence, and a few were fixed between susceptible and resistant strains. Presented in Table 3 are six nonsynonymous SNPs, one of which is a two nucleotide base deletion (SNP5), fixed between resistant and susceptible strains and the predicted amino acid change. The predicted location of these SNPs on the *ABCC2* protein are presented in Fig. 1. SNP4 resulted in an amino acid change from glycine in the susceptible strains to serine in the resistant strains. This change is predicted to be on ECL4 (Fig. 1). The two nucleotide base deletion (SNP5) induced a frameshift and a premature stop codon at amino acid 982, truncating the protein and resulting in the loss of the 11th and 12th transmembrane helices and NBD2 (Fig. 1).

**Table 3:** Allelic differences in the *ABCC2* gene and the predicted amino acid changes between Cry1Fa-susceptible (Cry1Fa-S) and Cry1Fa-resistant (Cry1Fa-R) *Ostrinia nubilalis* strains and the presence of the documented allelic differences in the laboratory-derived Cry1Fa-resistant strain.

Mutation name	Cry1Fa-S allele	Cry1Fa-R allele^1^	Amino acid change (S–R)	Present in laboratory-derived Cry1Fa-resistant strain^2^
SNP1	G	A	Glycine $\rightarrow$ Serine	Yes
SNP2	A	G	Asparagine $\rightarrow$ Aspartate	Yes
SNP3	A	T	Glutamate $\rightarrow$ Valine	Yes
SNP4	G	A	Glycine $\rightarrow$ Serine	Yes
SNP5	AT	DEL	Protein truncation	No
SNP6	G	C	Glycine $\rightarrow$ Alanine	Yes
SNPV^3^	C	A	Tyrosine $\rightarrow$ STOP	–

^1^Cry1Fa-R alleles present in either field-evolved, laboratory-derived, or both Cry1Fa-resistant strains.

^2^Based on available data obtained from Vellichirammal et al. (2015).

^3^SNPV denotes a mutational change present in the laboratory-derived Cry1Fa-resistant strain but not in the field-derived Cry1Fa-resistant strains.

The sequenced data were compared to the publicly available sequence of the laboratory-derived Cry1Fa-resistant *O. nubilalis* described in Vellichirammal et al.^18^ where we found 18 of the 20 identified SNPs present between the susceptible and Cry1Fa-resistant strains. Of the nonsynonymous SNPS, all five were fixed between the susceptible and laboratory-derived Cry1Fa-resistant strains (Table 3). However, SNP5 documented in our field-evolved Cry1Fa-resistant strains was not present in the laboratory-derived Cry1Fa-resistant *O. nubilalis* described in Vellichirammal et al.^18^ (Table 3). A non-synonymous SNP downstream of SNP6 was present in the laboratory-derived Cry1Fa-resistant strain described in Vellichirammal et al.^18^ (hereinafter referred to as SNPV) that resulted in protein truncation due to a premature stop codon, with the predicted loss of the NBD2 (Table 3).

### Genotyping assays

A restriction enzyme digest genotyping assay was developed to detect the six non-synonymous SNPs found in the sequenced resistant strains. The Cry1Fa-resistant strains were homozygous for the six non-synonymous SNPs, while the non-synonymous SNPs were only detected at a frequency of 0.04 in ON-S1 and 0.00 for ON-S2 (Table 4). BC-R was also homozygous for the six non-synonymous SNPs (Table 4).

**Table 4:** Allele frequencies of six non-synonymous SNPs associated with field-evolved Cry1Fa-resistance in Cry1Fa-susceptible, Cry1Fa-resistant, and back-crossed strains of *Ostrinia nubilalis* using digest enzymes.

Strain	n^1^	MspI^2^	Sau3AI^2^	HpyCH4IV^2^	BfaI^2^	BcoDI^2^	BsrDI^2^
ON-S1	25	0.04	0.04	0.04	0.04	0.04	0.04
ON-S2	25	0.00	0.00	0.00	0.00	0.00	0.00
NS-R	25	1.00	1.00	1.00	1.00	1.00	1.00
QC-R	25	1.00	1.00	1.00	1.00	1.00	1.00
BC-R^3^	25	1.00	1.00	1.00	1.00	1.00	1.00

^1^Total number of individuals genotyped.

^2^Cut sites are described in Table S3.

^3^BC-R represents a back-crossed Cry1Fa-resistant strain that is 96% genetically similar to ON-S1 but contains the Cry1Fa-resistant trait from crossing with NS-R.

**Figure 2:**
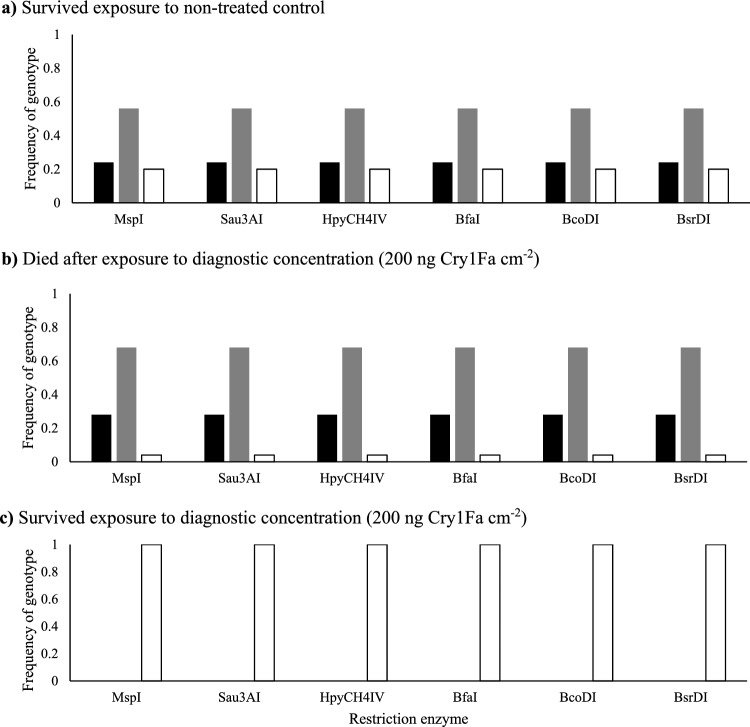
Allele frequencies of six non-synonymous SNPs associated with field-evolved Cry1Fa-resistance in *Ostrinia nubilalis* for the (**a**) survived exposure to non-treated control, (**b**) died after exposure to diagnostic concentration (200 ng Cry1Fa cm^−2^), and (**c**) survived exposure to diagnostic concentration (200 ng Cry1Fa cm^−2^) of the F2BC-RxON-S1 as determined using restriction enzyme digests. Black, grey, and white bars indicate homozygotes for the alleles associated with Cry1Fa susceptibility (p^2^), heterozygotes (2pq), and homozygotes for the alleles associated with Cry1Fa resistance (q^2^), respectively.

Randomly selected individuals from the last back-cross assay were genotyped and digest results are presented in Fig. 2. Restriction enzyme digests of randomly selected individuals from the last back-cross assay showed that individuals who survived exposure to non-treated control were in Hardy–Weinberg equilibrium (χ^2^ (2, n = 25) = 0.4400, P = 0.8025), with 24% homozygous for the six non-synonymous SNPs associated with Cry1Fa susceptibility (p^2^), 52% heterozygous (2pq), and 24% homozygous for the six non-synonymous SNPs (q^2^) (Fig. 2a). The percentage of genotyped individuals that died after exposure to diagnostic concentration were 56, 40, and 4% for homozygous susceptible (p2), heterozygous (2pq), and homozygous resistant (q^2^), respectively (Fig. 2b), which deviated from Hardy–Weinberg equilibrium (χ^2^ (2, n = 25) = 6.1200, P = 0.0469). Individuals that survived exposure to diagnostic concentration were 100% homozygous for the six non-synonymous SNPs (q^2^) (Fig. 2c).

## Discussion

Field-evolved Cry1Fa-resistance in *O. nubilalis* is closely linked to mutations in the *ABCC2* gene which functions as a receptor for the Cry1Fa toxin in susceptible insects. This is the first study to describe mutations linked to Bt resistance in *O. nubilalis*. Analogous comparisons between laboratory-derived and field-evolved Cry1F resistant *O. nubilalis* show striking similarities at the gene-level and with some of the detected mutations. Genotyping results using a Cry1Fa-resistant back-crossed strain provide support for linkage of six mutations to field-evolved Cry1Fa-resistance in *O. nubilalis*. The linkage tests detected no heterozygote survival among the back-crossed larvae exposed to the Cry1Fa toxin, suggesting that the resistance trait is completely recessive. Survival of approximately 25% of back-crossed individuals exposed to a diagnostic dose of Cry1Fa indicates that the resistance trait is controlled by a single gene. This result is similar to that reported within a laboratory-derived Cry1Fa-resistant *O. nubilalis* strain^17^.

A single nucleotide substitution that resulted in an amino acid change in ECL4 was present among the field-derived resistant strains in this study and in the laboratory-derived Cry1Fa-resistant strain^18^. ECL4 has been suggested as a potential binding site for Cry1 toxins as studies have shown that mutations in ECL4 confer resistance to Cry1 toxins in *S. frugiperda*, *S. exigua*, *Helicoverpa armigera* (Hübner), and *B. mori*^13–16,24,26,27^. However, the mutation described in this study requires further investigation to better indicate its link to the binding site of Cry1Fa on the *ABCC2* protein.

Another mutation described in this study is a two nucleotide base deletion that induces a frameshift, resulting in a premature stop codon, truncating the protein, and resulting in the loss of the last third of the second transmembrane domain including the NBD2. These nucleotide deletions were also present among the BC-R and QC-R strains tested, but not in the laboratory-derived Cry1Fa-resistant strain. However, SNPV was present in the laboratory-derived Cry1Fa-resistant strain and also resulted in protein truncation, with the predicted loss of the NBD2. High-level resistance to Cry1Fa was documented using a CRISPR-mediated *ABCC2* gene knockout that resulted in the loss of both ECL4 and NBD2 in *O. furnacalis* and *S. exigua*^23,28^. Furthermore, mutational changes associated with field-evolved Cry1Fa-resistant *S. frugiperda* from Puerto Rico and Florida resulted in *ABCC2* protein truncation with the loss of the second transmembrane domain, including ECL4 and NBD2^6,7^. Mutations in NBD2, including the NBD2-deletion, did not, on their own, alter Cry1Fa susceptibility in baculovirus-free insect cell expression of *ABCC2* variants of *S. frugiperda*^13^. Using functional genetic testing such as baculovirus-free insect cells expressing the mutations documented in this study, either alone or in combination, can help determine the mutations responsible for Cry1Fa-resistance in *O. nubilalis* and determine the mechanisms behind Cry1Fa resistance.

*ABCC2* has been described as a binding receptor for Cry1Ab, Cry1Ac, and Cry1A.105, in addition to Cry1Fa^15,16,29^. Mutations to *ABCC2* resulting in Cry1Fa-resistance may also alter *O. nubilalis* susceptibility to other Cry1 toxins if resistance was due to an alteration of the shared binding sites^30,31^. CRISPR-mediated *ABCC2* gene knockout in *O. furnacalis* resulted in over 300-fold resistance to Cry1Fa and 4–eightfold resistance to Cry1Ab and Cry1Ac^23^. Knocking out *ABCC2* in *S. frugiperda* conferred over 120-fold resistance to Cry1Fa and Cry1Ab^32^. In a cell expression study of *ABCC2* mutations documented in *S. frugiperda*, single nucleotide substitutions in ECL4 conferred over 630-fold resistance to Cry1Fa and over ninefold resistance to Cry1Ab and Cry1A.105^13^. *ABCC2* as a shared receptor among Cry1 toxins was also documented in other Lepidopteran species such as *B. mori, H. armigera,* and *S. exigua*^15,16,22,24,28^.

The frequency of the Cry1Fa resistance allele before the adoption of Cry1Fa corn in NS is unknown as Bt resistance monitoring was not conducted in the Maritimes region before 2018. The Cry1Fa resistance allele frequency (0.04) observed in the ON-S1 strain in the present study is greater than that reported in the U.S. corn belt (> 10^–3^)^33^. The Cry1Fa resistance allele frequency in U.S. corn belt was estimated using an F_1_, where field-collected individuals were crossed with a resistant laboratory strain, and F_2_ screening approach to estimate Cry1Fa resistance allele frequency. The results revealed the presence of the Cry1Fa resistance allele frequency at higher than estimated levels even during the first year of commercialization of Cry1Fa corn; however, there was no indication of that frequency increasing over the 7 years of the study^33^. The origin of this allele is also unclear. However, *B. thuringiensis* is ubiquitous in the environment^34^ and contact with this bacterium throughout *O. nubilalis* evolution may have resulted in the presence of alleles conferring resistance to Bt toxins^3^. Evolution of resistance is likely to capitalize on alleles already present in the population^3^.

The analogous comparison of the genetic basis of Bt resistance between laboratory-derived and field-evolved Cry1Fa resistance in *O. nubilalis* show a great deal of similarities at both the gene-level and with some of the detected mutations. To our knowledge, only one other study conducted analogous comparisons between laboratory-derived and field-evolved Bt resistance, finding striking similarities between the two strains of *P. gossypiella*^9^. These similarities highlight the usefulness of laboratory selection for identifying genes important in field-evolved Bt resistance, but may not necessarily capture the specific mutations in those genes. The present study is the first to describe mutations in *O. nubilalis* linked to Bt resistance and is one of only three studies to identify molecular genetic basis of practical Bt resistance. Using the genotyping method described in this study, changes in the frequency of Cry1Fa-resistance across the range of *O. nubilalis* can be detected, allowing for a more rapid implementation of Bt resistance management strategies.

## Methods and material

### Insect strains

Strains of *O. nubilalis* used in this study were initiated from fifth-instar larvae collected from commercial corn fields. Cry1Fa-susceptible strains were collected from Winger, ON (ON-S2) and Delaware, ON (ON-S1) and in 2010 and 2016, respectively. Cry1Fa-resistant strains were collected from Masstown, NS (NS-R) and Saint-Mathieu-de-Boloeil, QC (QC-R) in 2019^12^ (*SI Appendix*, Table S1). Larvae were reared in 36 cm diameter plastic tubs containing meridic diet with corrugated cardboard rings placed above the diet surface as described by Smith et al.^11^. Once the majority of larvae had pupated in the rings, they were transferred to cages (30 cm × 30 cm × 60 cm) with waxed paper sheets placed on top of the mesh roof for oviposition. Waxed sheets were replaced daily and placed into plastic sweater boxes lined with moistened paper towels. All rearing stages and bioassays were maintained at 26:18 °C, 60% RH, and 16:8 (L:D) h photoperiod.

### Toxin-overlay diet bioassays

The susceptibility of *O. nubilalis* strains to Cry1Fa was determined using toxin-overlay diet bioassays. Diet was prepared in the same manner as for rearing^11^. Using a repeater pipette, 1 mL of diet was dispensed into each well of a 128-well bioassay tray (Bio-16, CD International, Pitman, NJ) resulting in a surface area of 2.0 cm^2^. After the diet has solidified, wells were covered with adhesive ventilated lids (Frontier Agricultural Sciences, Newark, DE), and stored at 4 °C. Lyophilized toxin standard containing > 95% purity of activated Cry1Fa, produced from *Escherichia coli* (Migula) containing the Cry1F gene, was obtained from M. Pusztai-Carey (Case-Western University, Cleveland, OH), and stored at −80 °C. Cry1Fa toxin was constituted using 10 mM CAPS (3-cyclohexylamino-1-propane sulfonic acid) buffer solution with the pH adjusted to 10.5 using 10 N NaOH. Cry1Fa toxin solution was diluted using 10 mM CAPS buffer solution to a diagnostic concentration of 200 ng cm^−2^. The non-treated control was treated with 10 mM CAPS buffer solution only. The diagnostic concentration of Cry1Fa or the non-treated control solution was applied to the diet surface of each well in 30 μl aliquots using a repeater pipette. Trays were tilted in all directions to cover the entire diet surface with solution. Trays were kept in a fume hood as the solvent component of the solution evaporated. A single neonate (< 24 h old) larva was transferred with a fine-paint paint brush to each well of the bioassay tray. Infested wells were covered with adhesive lids, placed into rearing conditions, and covered with cardboard to prevent condensation. A total of 24 larvae were treated per solution per bioassay. Individual larval mortality and weight were recorded after seven days. Stunted larvae that were alive but weighed less than 0.1 mg were considered dead. Each bioassay was repeated five times for each strain.

### Introgression experiment

Adults from ON-S1 and NS-R were crossed following the cage method described earlier in the following configurations: 50 ON-S1♀ × 50 NS-R♂ and 50 NS-R♀ × 50 ON-S1♂. Progeny (F_1_) of the crosses were allowed to mate and their offspring (F_2_) were exposed to a diagnostic concentration of Cry1Fa as described for the toxin-overlay diet bioassays. Survivors of Cry1Fa exposure were then back-crossed to the ON-S1 strain. This process was repeated five times resulting in a Cry1Fa-resistant strain (BC-R) that is > 96% genetically similar to the ON-S1 strain. Toxin-overlay diet bioassays were conducted with the BC-R strain as described above with 24 and 72 larvae exposed to the zero and diagnostic concentration of Cry1Fa, respectively, and replicated five times. Restriction enzyme genotype analysis was conducted on 25 randomly selected larvae from all five replications from each of the following categories: survived exposure to 0 ng Cry1Fa cm^−2^, died after exposure to 200 ng Cry1Fa cm^−2^, and survived exposure to 200 ng Cry1Fa cm^−2^.

### Sequencing

To determine single nucleotide polymorphisms (SNPs) in the *ABCC2* gene associated with susceptibility and resistance to the Cry1Fa toxin, RNA samples from two ON-S1 and two NS-R individuals were sequenced. RNA was extracted using TRIzol™ Plus RNA purification kit (Invitrogen™, Waltham, MA) following manufacturer’s instructions. Samples were amplified with SuperScript™ IV One-Step RT-PCR System kit (Invitrogen™, Waltham, MA) on a VeritiPro™ Thermal Cycler (Applied Biosystems™, Waltham, MA) and primers were designed as described below to amplify the target locus. The cycling conditions included reverse transcription at 50 °C for 10 m, followed by initial denaturation at 98 °C for 2 m, then 40 cycles at 98 °C for 10 s, 55 °C for 10 s, and 72 °C for 1 m, and a final extension at 72 °C for 5 min. Since the sequence for the *O. nubilalis ABCC2* gene is unknown, we used the sequence from a closely related species, *O. furnacalis* (NCBI Accession #MN783372) to search the *O. nubilalis* draft genome (NCBI Assembly ASM892168v1). Search results via BLAST provided a single genomic location for *ABCC2* on scaffold SWFO01000764.1 at positions 115,342–132,146 (16,804 bp). PCR primers were developed to amplify the coding region (cDNA) at this locus in 5 separate but overlapping amplicons ranging from 774–918 bp in length (*SI Appendix*, Table S2). Coding used to obtain the ECB *ABCC2* gene is available at https://github.com/mikesovic/Farhan_etal_ABCC2. PCR products were loaded into 1.5% agarose gels and separated at 100 V for 1 h. Resulting fragments sizes were estimated by comparison to a DirectLoad™ 50-bp ladder (Sigma-Aldrich, St. Louis, MO). Amplicons were sent to the Advanced Analysis Centre (University of Guelph, Guelph, ON) for Sanger sequencing. Sequence data were aligned using BioEdit 7.2, to the *O. nubilalis ABCC2* gene and SNPs consistent between ON-S1 and NS-R individuals were documented. Sequencing data were compared to the publicly available sequences of the laboratory-derived Cry1Fa-resistant *O. nubilalis* described in Vellichirammal et al.^18^.

Open reading frame (ORF) and amino acid sequence of the *ABCC2* protein were predicted using the ORF finder software (https://www.ncbi.nlm.nih.gov/orffinder/) and ExPASy Bioinformatics resource portal (http://web.expasy.org/translate/), respectively. The transmembrane topology was predicted using the Phobius web server^35^. BLAST search of the NCBI Conserved Domains Database (http://www.ncbi.nlm.nih.gov/cdd) was used to predict the ATP binding cassettes.

### Genotyping assay

Based on the sequencing data, a restriction enzyme digest method was developed to determine the presence and linkage of SNPs in resistant individuals. Genomic DNA was extracted from fifth instars of the ON-S1, ON-S2, NS-R, and QC-R strains, as well as from first instars of the ON-S1 × NS-R and BC-R strains using QuickExtract™ DNA Extraction Solution (Lucigen Corporation, Middleton, WI) following the manufacturer’s protocol and quantified using Ultraspec 2100 pro spectrometer (Biochrom Ltd, Cambridge, UK). Samples were amplified using GoTaq G2 Green Master Mix (Promega, Madison, WI), with forward and reverse primers (Sigma-Aldrich, St. Louis, MO) (*SI Appendix*, Table S3) using VeritiPro™ Thermal Cycler (Applied Biosystems™, Waltham, MA). The cycling conditions included initial denaturation at 95 °C for 5 m, then 37 cycles at 95 °C for 30 s, 58 °C for 30 s, and 72 °C for 40 s, and a final extension at 72 °C for 5 min. The amplicons were digested using the restriction enzymes listed in *SI Appendix*, Table S3 (New England Biolab, Whitby, ON) for 1.5 h following the manufacturer’s protocol. Five of these restriction enzymes cut the resistant allele at the indicated position, while enzyme MspI cut the susceptible allele at SNP1 (*SI Appendix*, Table S3). Digest products were loaded on 1.5% agarose gel and separated using 100 V for 1 h. The resulting fragment sizes were analysed using a DirectLoad™ 50-bp ladder (Sigma-Aldrich, St. Louis, MO). Protocol describing the PCR and digest methods is available in *SI Appendix*.

### Data analyses

#### Toxin-overlay diet bioassays

Mortality at the diagnostic concentration of Cry1Fa was analysed using PROC GLIMMIX in SAS v. 9.4 (SAS Institute, Cary, NC) with strain as the fixed effect and replicate as a random effect. Mortality data followed a beta distribution with a logit link and values of 0 and 1 were replaced with 0.0001 and 0.9999, respectively^36^. Tukey’s Honestly Significant Difference (HSD) test was used for multiple treatment comparisons and the α level for statistical significance was set at 0.05.

#### Genotyping assay

A chi-squared test was conducted using PROC FREQ in SAS v. 9.4 (SAS Institute, Cary, NC) to determine if the genotypes for the individuals tested in the back-cross experiment were in Hardy–Weinberg equilibrium.

## Supplementary Information

Supplementary Information.

## Data Availability

The data generated and/or analyzed in the current study are available from the corresponding author on reasonable request.
