# Supplementary material for: Genetic mutations linked to field‐evolved Cry1Fa-resistance in the European corn borer, Ostrinia nubilalis
Source: Sci Rep. 2023 May 18;13:8081. doi: 10.1038/s41598-023-35252-y (PMC10195855; doi:10.1038/s41598-023-35252-y)
Supplement: Supplementary file 1 — Supplementary Information. [file 41598_2023_35252_MOESM1_ESM.docx]

Scientific Reports Supplemental information:

**Genetic mutations linked to field‐evolved Cry1Fa-resistance in the European corn borer, *Ostrinia nubilalis***

Yasmine Farhan*, Jocelyn L. Smith, Michael G. Sovic2 and Andrew P. Michel

*Email: [yfarhan@uoguelph.ca](mailto:yfarhan@uoguelph.ca)

Table S1. Location and collection dates of *Ostrinia nubilalis* strains in Canada used in the study.

| Strain | Cry1Fa susceptibility | Collection location  (Nearest town, Province) | # of initial collection | Date of collection | Generation tested |
| --- | --- | --- | --- | --- | --- |
| ON-S1 | Susceptible | Delaware, Ontario | 49 | 06-Nov-16 | F29 |
| ON-S2 | Susceptible | Winger, Ontario | 41 | 22-Nov-10 | F112 |
| NS-R1 | Resistant | Masstown, Nova Scotia | 38 | 04-Sep-19 | F6 |
| QC-R1 | Resistant | Saint-Mathieu-de-Beloeil, Quebec | 28 | 13-Nov-19 | F2 |

Table S2. Oligonucleotide primer pairs that anneal to exon regions of the ABCC2 gene of *Ostrinia nubilalis* and the fragment lengths (bp) produced after PCR amplification.

| Name | Oligonucleotide primer | Fragment length (bp) |
| --- | --- | --- |
| Pset-1F | 5'- TCGTTAAAGAGACCAGTCCA -3' | 918 |
| Pset-1R | 5'- CTGCATTTCGTAGGCTCG -3' |  |
| Pset-2F | 5'- AATAACGCCAATACAGCAGG -3' | 905 |
| Pset-2R | 5'- GCTCTCCAACCAAAGACAGA -3' |  |
| Pset-3F | 5'- GCTGCCGTATGACTCTGAAA -3' | 909 |
| Pset-3R | 5'- TGAAGACCATGTCGTGGATA -3' |  |
| Pset-4F | 5'- TACCTATGGGTGTACGGTGG -3' | 918 |
| Pset-4R | 5'- TGATCTGAACATTCAAATTCTTG -3' |  |
| Pset-5F | 5'- ACAAACCTGGCCTACCAGT -3' | 774 |
| Pset-5R | 5'- GGCCATTTCAAACAGCACTT -3' |  |

Table S3. Oligonucleotide primer pairs that anneal to the ABCC2 gene of *Ostrinia nubilalis* for PCR amplification of regions that contain non-synonymous single nucleotide polymorphisms (SNPs) and two nucleotide deletions.

| Name | Oligonucleotide primer | SNP | Restriction enzyme | Allele specific | Fragment length (bp) |
| --- | --- | --- | --- | --- | --- |
| SNP1-F | 5'- TCGTTAAAGAGACCAGTCCA -3' | G | MspI(+) | Cry1F-S | 233, 133 |
| SNP1-R | 5'- CAAACACTGCCAATTCAAAGC -3' | A | MspI(-) | Cry1F-R | 366 |
| SNP2-F^1^ | 5'- AACATGGCTCTTGTGGGGC -3' | A | Sau3AI(-) | Cry1F-S | 222 |
| SNP2-R^1^ | 5'- AAAGGCGCCCGGCATAT -3' | G | Sau3AI(-) | Cry1F-R | 139, 83 |
| SNP3-F^1^ | 5'- AACATGGCTCTTGTGGGGC -3' | A | HpyCH4IV(-) | Cry1F-S | 222 |
| SNP3-R^1^ | 5'- AAAGGCGCCCGGCATAT -3' | T | HpyCH4IV(+) | Cry1F-R | 155, 67 |
| SNP4-F | 5'- CCTGAAGATCAAGAGCCTGGT -3' | G | BfaI(-) | Cry1F-S | 322 |
| SNP4-R | 5'- TGAAGACCATGTCGTGGATA -3' | A | BfaI(+) | Cry1F-R | 167, 155 |
| SNP5-F | 5'- CGTGCTGGTGATAAACGCG -3' | AT | BcoDI(-) | Cry1F-S | 774 |
| SNP5-R | 5'- AACCCCCGGTTGCTAAGT -3' | DEL | BcoDI(+) | Cry1F-R | 507, 265 |
| SNP6-F | 5'- ACTGGTAGGCCAGGTTTGT -3' | G | BsrDI(-) | Cry1F-S | 496 |
| SNP6-R | 5'- TGTACCGTACTGTTGCCG -3' | C | BsrDI(+) | Cry1F-R | 301, 186 |

^1^ SNP2-F/R use the same oligonucleotide primer sequence to SNP3-F/R, respectively.

**PCR Protocol**

1. Combine 9.5 uL of water, 1 uL of forward primer (refer to table S3), 1 uL of reverse primer (refer to table S3), and 12.5 uL of PCR Mastermix in a microcentrifuge tube and mix gently.

2. Place the tube on ice and add 1 uL of DNA template.

3. Place the tube in a thermal cycler and program it for the following protocol:

• Initial Denaturation: 95°C for 5 minutes

• Cycle – 32

Denaturation: 95°C for 30 seconds

Annealing: 58°C for 30 seconds

Extension: 72°C for 1 minute

• Final Extension: 72°C for 5 minutes

4. Remove the tube from the thermal cycler and place on ice.

5. Prepare a 1.5% agarose gel (1X 2.25 g in 150 ul TBE buffer with 1X 7.5 ul EtBr)

6. Load 5 uL of product, 5 uL of ladder into the wells of the gel and run for 1 h at 100V.

7. Interpret results using table S3

**Digest Protocol**

1. Combine 20 uL of distilled water, 2.5 uL of buffer (refer to table below for buffer type), 0.5 uL od digest enzyme (refer to table below for type), and 2 uL of amplicon DNA into a microcentrifuge tube and mix gently

2. Incubate for 1.5 h at 37C (65C for BsrDI digest enzyme)

3. Prepare a 1.5% agarose gel (1X 2.25 g in 150 ul TBE buffer with 1X 7.5 ul EtBr)

4. Load 15 uL of product, 5 uL of ladder into the wells of the gel and run for 1 h at 100V

5. Interpret results using table S3

| Components | SNP1 | SNP2 | SNP3 | SNP4 | SNP5(AT) | SNP6 |
| --- | --- | --- | --- | --- | --- | --- |
| Digest enzyme | MspI | Sau3AI | HpyCH4IV | BfaI | BcoDI – | BsrDI |
| Water (uL) | 20 | 20 | 20 | 20 | 20 | 20 |
| Buffer type | RCutsmart | RCutsmart | R1.1 | RCutsmart | RCutsmart | R2.1 |
|  |  |  | RCutsmart |  |  |  |
| 10X NEBuffer (uL) | 2.5 | 2.5 | 2.5 | 2.5 | 2.5 | 2.5 |
| Digest enzyme (uL) | 0.5 | 0.5 | 0.5 | 0.5 | 0.5 | 0.5 |
| DNA (uL) amplicon | 2 | 2 | 2 | 2 | 2 | 2 |
